# Oscillation and variation inequalities for the multilinear singular integrals related to Lipschitz functions

**DOI:** 10.1186/s13660-017-1568-8

**Published:** 2017-11-25

**Authors:** Yue Hu, Yueshan Wang

**Affiliations:** 1 College of Mathematics and Informatics, Henan Polytechnic University, Jiaozuo, 454003 China; 2 Department of Mathematics, Jiaozuo University, Jiaozuo, 454003 China

**Keywords:** 42B25, 42B20, 47G10, oscillation operator, variation operator, multilinear operator, Lipschitz function

## Abstract

The main purpose of this paper is to establish the weighted $(L^{p},L^{q})$ inequalities of the oscillation and variation operators for the multilinear Calderón-Zygmund singular integral with a Lipschitz function.

## Introduction and results

Let *K* be a kernel on $\mathbb{R}\times\mathbb{R}\setminus\{ (x,x): x\in\mathbb{R}\}$. Suppose that there exist two constants *δ* and *C* such that

1.1 $$\begin{aligned} &\bigl\vert K(x,y) \bigr\vert \leq\frac{C}{ \vert x-y \vert }\quad \mbox{for } x\neq y; \end{aligned}$$

1.2 $$\begin{aligned} &\bigl\vert K(x,y)-K\bigl(x',y\bigr) \bigr\vert \leq\frac{C \vert x-x' \vert ^{\delta}}{ \vert x-y \vert ^{1+\delta}}\quad \mbox{for } \vert x-y \vert \geq2 \bigl\vert x-x' \bigr\vert ; \end{aligned}$$

1.3 $$\begin{aligned} &\bigl\vert K(x,y)-K\bigl(x,y'\bigr) \bigr\vert \leq\frac{C \vert y-y' \vert ^{\delta}}{ \vert x-y \vert ^{1+\delta}} \quad \mbox{for } \vert x-y \vert \geq2 \bigl\vert y-y' \bigr\vert . \end{aligned}$$

We consider the family of operators $T=\{T_{\epsilon}\}_{\epsilon>0}$ given by

1.4 $$\begin{aligned} T_{\epsilon}f(x)= \int_{ \vert x-y \vert >\epsilon}K(x,y) f(y)\,dy. \end{aligned}$$

A common method of measuring the speed of convergence of the family $T_{\epsilon}$ is to consider the square functions

$$\begin{aligned} \Biggl(\sum_{i=1}^{\infty} \vert T_{\epsilon_{i}}f-T_{\epsilon _{i+1}}f \vert ^{2} \Biggr)^{1/2}, \end{aligned}$$

where $\epsilon_{i}$ is a monotonically decreasing sequence which approaches 0. For convenience, other expressions have also been considered. Let $\{t_{i}\}$ be a fixed sequence which decreases to zero. Following [1], the oscillation operator is defined as

$$\begin{aligned} \mathcal{O}(Tf) (x)= \Biggl(\sum_{i=1}^{\infty}\sup_{t_{i+1}\leq \epsilon_{i+1}< \epsilon_{i}\leq t_{i}} \bigl\vert T_{\epsilon _{i+1}}f(x)-T_{\epsilon_{i}}f(x) \bigr\vert ^{2} \Biggr)^{1/2} \end{aligned}$$

and the *ρ*-variation operator is defined as

$$\begin{aligned} \mathcal{V}_{\rho}(Tf) (x)=\sup_{\epsilon_{i}\searrow0 } \Biggl(\sum _{i=1}^{\infty}\bigl\vert T_{\epsilon_{i+1}}f(x)-T_{\epsilon_{i}}f(x) \bigr\vert ^{\rho}\Biggr)^{1/\rho}, \end{aligned}$$

where the sup is taken over all sequences of real number $\{ \epsilon_{i}\}$ decreasing to zero.

The oscillation and variation for some families of operators have been studied by many authors on probability, ergodic theory, and harmonic analysis; see [2–4]. Recently, some authors [5–8] researched the weighted estimates of the oscillation and variation operators for the commutators of singular integrals.

Let *m* be a positive integer, let *b* be a function on $\mathbb{R}$, and let $R_{m+1}(b;x,y)$ be the $m+1$th Taylor series remainder of *b* at *x* expander about *y*, *i.e.*

$$\begin{aligned} R_{m+1}(b;x,y)=b(x)-\sum_{\alpha\leq m} \frac{1}{\alpha!} b^{(\alpha )}(y) (x-y)^{\alpha}. \end{aligned}$$

We consider the family of operators $T^{b}=\{T^{b}_{\epsilon}\}_{\epsilon >0}$, where $T^{b}_{\epsilon}$ are the multilinear singular integral operators of $T_{\epsilon}$,

1.5 $$\begin{aligned} T^{b}_{\epsilon}f(x)= \int_{ \vert x-y \vert >\epsilon}\frac{ R_{m+1}(b;x,y)}{ \vert x-y \vert ^{m}}K(x,y)f(y)\,dy. \end{aligned}$$

Note that when $m=0$, $T^{b}_{\epsilon}$ is just the commutator of $T_{\epsilon}$ and *b*, which is denoted by $T_{\epsilon,b}$, that is to say

1.6 $$\begin{aligned} T_{\epsilon,b} f(x)= \int_{ \vert x-y \vert >\epsilon}\bigl(b(x)-b(y)\bigr)K(x,y)f(y)\,dy. \end{aligned}$$

However, when $m>0$, $T^{b}_{\epsilon}$ is a non-trivial generation of the commutator. It is well known that multilinear operators are of great interest in harmonic analysis and have been widely studied by many authors (see [9–13]).

A locally integrable function *b* is said to be in Lipschitz space $\mathrm{Lip}_{\beta}(\mathbb{R})$ if

$$\begin{aligned} \Vert b \Vert _{\dot{\wedge}_{\beta}}=\sup_{I}\frac{1}{ \vert I \vert ^{1+\beta}} \int _{I} \bigl\vert b(x)-b_{I} \bigr\vert \,dx< \infty, \end{aligned}$$

where

$$\begin{aligned} b_{I}=\frac{1}{ \vert I \vert } \int_{I}b(x)\,dx. \end{aligned}$$

In this paper, we will study the boundedness of oscillation and variation operators for the family of the multilinear singular integral related to a Lipschitz function defined by (1.5) in weighted Lebesgue space. Our main results are as follows.

### Theorem 1.1

*Suppose that* $K(x,y)$ *satisfies* (1.1)-(1.3), $b^{(m)}\in\dot{\wedge}_{\beta}$, $0<\beta\leq\delta<1$, *where* *δ* *is the same as in* (1.2). *Let* $\rho>2$, $T=\{T_{\epsilon}\} _{\epsilon>0}$ *and* $T^{b}=\{T^{b}_{\epsilon}\}_{\epsilon>0}$ *be given by* (1.4) *and* (1.5), *respectively*. *If* $\mathcal{O}(T)$ *and* $\mathcal{V}_{\rho}(T)$ *are bounded on* $L^{p_{0}}(\mathbb {R},dx)$ *for some* $1< p_{0}<\infty$, *then*, *for any* $1< p<1/\beta$ *with* $1/q=1/p-\beta$, $\omega\in A_{p,q}(\mathbb{R})$, $\mathcal{O}(T^{b})$ *and* $\mathcal{V}_{\rho}(T^{b})$ *are bounded from* $L^{p}(\mathbb{R},\omega^{p} \,dx)$ *into* $L^{q}(\mathbb{R},\omega^{q} \,dx)$.

### Corollary 1.1

*Suppose that* $K(x,y)$ *satisfies* (1.1)-(1.3), $b\in\dot{\wedge}_{\beta}$, $0<\beta\leq\delta<1$, *where* *δ* *is the same as in* (1.2). *Let* $\rho>2$, $T=\{T_{\epsilon}\} _{\epsilon>0}$ *and* $T_{b}=\{T_{b,\epsilon}\}_{\epsilon>0}$ *be given by* (1.4) *and* (1.6), *respectively*. *If* $\mathcal{O}(T)$ *and* $\mathcal{V}_{\rho}(T)$ *are bounded on* $L^{p_{0}}(\mathbb {R},dx)$ *for some* $1< p_{0}<\infty$, *then*, *for any* $1< p<1/\beta$ *with* $1/q=1/p-\beta$, $\omega\in A_{p,q}(\mathbb{R})$, $\mathcal{O}(T_{b})$ *and* $\mathcal{V}_{\rho}(T_{b})$ *are bounded from* $L^{p}(\mathbb{R},\omega^{p} \,dx)$ *into* $L^{q}(\mathbb{R},\omega^{q} \,dx)$.

In this paper, we shall use the symbol $A\lesssim B$ to indicate that there exists a universal positive constant *C*, independent of all important parameters, such that $A\leq CB$. $A\thickapprox B$ means that $A\lesssim B$ and $B\lesssim A$.

## Some preliminaries

### Weight

A weight *ω* is a nonnegative, locally integrable function on $\mathbb{R}$. The classical weight theories were introduced by Muckenhoupt and Wheeden in [14] and [15].

A weight *ω* is said to belong to the Muckenhoup class $A_{p}(\mathbb{R})$ for $1< p<\infty$, if there exists a constant *C* such that

$$\begin{aligned} \biggl(\frac{1}{ \vert I \vert } \int_{I}\omega(x)\,dx \biggr) \biggl(\frac {1}{ \vert I \vert } \int_{I}\omega(x)^{-\frac{1}{p-1}}\,dx \biggr)^{p-1}\leq C \end{aligned}$$

for every interval *I*. The class $A_{1}(\mathbb{R})$ is defined by replacing the above inequality with

$$\begin{aligned} \frac{1}{ \vert I \vert } \int_{I}\omega(x)\,dx\lesssim \mathop{\operatorname{ess}\operatorname{inf}}_{x\in I} w(x)\quad\mbox{for every ball } I\subset\mathbb{R}. \end{aligned}$$

When $p=\infty$, we define $A_{\infty}(\mathbb{R})=\bigcup_{1\leq p<\infty}A_{p}(\mathbb{R})$.

A weight $\omega(x)$ is said to belong to the class $A_{p,q}(\mathbb{R})$, $1< p\leq q<\infty$, if

$$\begin{aligned} \biggl(\frac{1}{ \vert I \vert } \int_{I}\omega(x)^{q}\,dx \biggr)^{1/q} \biggl(\frac {1}{ \vert I \vert } \int_{I}\omega(x)^{-p'}\,dx \biggr)^{1/p'}\leq C. \end{aligned}$$

It is well known that if $\omega\in A_{p.q}(\mathbb{R})$, then $\omega^{q}\in A_{\infty}(\mathbb{R})$.

### Function of $\mathrm{Lip}_{\beta}(\mathbb{R})$

The function of $\mathrm{Lip}_{\beta}(\mathbb{R})$ has the following important properties.

#### Lemma 2.1

*Let* $b\in \mathrm{Lip}_{\beta}(\mathbb{R})$. *Then*

1. $1\leq p<\infty$
  $$\begin{aligned} \sup_{I}\frac{1}{ \vert I \vert ^{\beta}} \biggl(\frac{1}{ \vert I \vert } \int _{I} \bigl\vert b(x)-b_{I} \bigr\vert ^{p}\,dx \biggr)^{1/p}\leq C \Vert b \Vert _{\dot{\wedge}_{\beta}}; \end{aligned}$$
2. *for any* $I_{1}\subset I_{2}$,
  $$\begin{aligned} \frac{1}{ \vert I_{2} \vert } \int_{I_{2}} \bigl\vert b(y)-b_{I_{1}} \bigr\vert \,dy \lesssim\frac { \vert I_{2} \vert }{ \vert I_{1} \vert } \vert I_{2} \vert ^{\beta} \Vert b \Vert _{\dot{\wedge}_{\beta}}. \end{aligned}$$

### Maximal function

We recall the definition of Hardy-Littlewood maximal operator and fractional maximal operator. The Hardy-Littlewood maximal operator is defined by

$$\begin{aligned} M(f) (x)=\sup_{I\ni x}\frac{1}{ \vert I \vert } \int_{I} \bigl\vert f(y) \bigr\vert \,dy. \end{aligned}$$

The fractional maximal function is defined as

$$\begin{aligned} M_{\beta,r}(f) (x)=\sup_{I\ni x} \biggl(\frac{1}{ \vert I \vert ^{1-r\beta}} \int _{I} \bigl\vert f(y) \bigr\vert ^{r}\,dy \biggr)^{1/r} \end{aligned}$$

for $1\leq r<\infty$. In order to simplify the notation, we set $M_{\beta}(f)(x)=M_{\beta,1}(f)(x)$.

#### Lemma 2.2

*Let* $1< p<\infty$ *and* $\omega\in A_{\infty}(\mathbb{R})$. *Then*

$$\begin{aligned} \Vert M f \Vert _{L^{p}(\omega)}\lesssim \bigl\Vert M^{\sharp}f \bigr\Vert _{L^{p}(\omega)} \end{aligned}$$

*for all* *f* *such that the left hand side is finite*.

#### Lemma 2.3

*Suppose* $0<\beta<1$, $1\leq r< p<1/\beta$, $1/q=1/p-\beta$. *If* $\omega\in A_{p,q}(\mathbb{R})$, *then*

$$\begin{aligned} \Vert M_{\beta,r} f \Vert _{L^{q}(\omega^{q})}\lesssim \Vert f \Vert _{L^{p}(\omega^{p})}. \end{aligned}$$

### Taylor series remainder

The following lemma gives an estimate on Taylor series remainder.

#### Lemma 2.4

[10] *Let* *b* *be a function on* $\mathbb{R}$ *and* $b^{(m)}\in L^{s}(\mathbb {R})$ *for any* $s>1$. *Then*

$$\begin{aligned} \bigl\vert R_{m}(b;x,y) \bigr\vert \lesssim \vert x-y \vert ^{m} \biggl(\frac{1}{ \vert I_{x}^{y} \vert } \int _{I_{x}^{y}} \bigl\vert b^{(m)}(z) \bigr\vert ^{s}\,dz \biggr)^{1/s}, \end{aligned}$$

*where* $I_{x}^{y}$ *is the interval* $(x-5 \vert x-y \vert , x+5 \vert x-y \vert )$.

### Oscillation and variation operators

We consider the operator

$$\begin{aligned} \mathcal{O}'(Tf) (x)= \Biggl(\sum_{i=1}^{\infty}\sup_{t_{i+1}< \delta _{i}< t_{i}} \bigl\vert T_{t_{i+1}}f(x)-T_{\delta_{i}}f(x) \bigr\vert ^{2} \Biggr)^{1/2}. \end{aligned}$$

It is easy to check that

$$\begin{aligned} \mathcal{O}'(Tf)\thickapprox\mathcal{O}(Tf). \end{aligned}$$

Following [4], we denote by *E* the mixed norm Banach space of two variable function *h* defined on $\mathbb{R}\times\mathbb{N}$ such that

$$\begin{aligned} \Vert h \Vert _{E}\equiv \biggl(\sum _{i} \Bigl(\sup_{s} \bigl\vert h(s,i) \bigr\vert \Bigr)^{2} \biggr)^{1/2}< \infty. \end{aligned}$$

Given $T=\{T_{\epsilon}\}_{\epsilon>0}$, where $T_{\epsilon}$ defined as (1.4), for a fixed decreasing sequence $\{t_{i}\}$ with $t_{i}\searrow0$, let $J_{i}=(t_{i+1},t_{i}]$ and define the *E*-valued operator $\mathcal{U}(T): f\rightarrow\mathcal{U}(T)f$ by

$$\begin{aligned} \mathcal{U}(T)f(x)=\bigl\{ T_{t_{i+1}}f(x)-T_{s}f(x)\bigr\} _{s\in J_{i},i\in\mathbb{N}}= \biggl\{ \int_{\{t_{i+1}< \vert x-y \vert < s\} }K(x,y)f(y)\,dy \biggr\} _{s\in J_{i},i\in\mathbb{N}}. \end{aligned}$$

Then

$$\begin{aligned} \mathcal{O}'(Tf) (x)={}& \bigl\Vert \mathcal{U}(T)f(x) \bigr\Vert _{E}= \bigl\Vert \bigl\{ T_{t_{i+1}}f(x)-T_{s}f(x) \bigr\} _{s\in J_{i},i\in\mathbb{N}} \bigr\Vert _{E} \\ ={}& \biggl\Vert \biggl\{ \int_{\{t_{i+1}< \vert x-y \vert < s\}}K(x,y)f(y)\,dy \biggr\} _{s\in J_{i},i\in\mathbb{N}} \biggr\Vert _{E}. \end{aligned}$$

On the other hand, let $\Theta=\{\beta: \beta=\{\epsilon_{i}\} ,\epsilon_{i}\in\mathbb{R},\epsilon_{i}\searrow0\}$. We denote by $F_{\rho}$ the mixed norm space of two variable functions $g(i,\beta)$ such that

$$\begin{aligned} \Vert g \Vert _{F_{\rho}}\equiv\sup_{\beta}\biggl(\sum _{i} \bigl\vert g(i,\beta) \bigr\vert ^{\rho}\biggr)^{1/\rho}. \end{aligned}$$

We also consider the $F_{\rho}$-valued operator $\mathcal {V}(T):f\rightarrow\mathcal{V}(T)f$ given by

$$\begin{aligned} \mathcal{V}(T)f(x)=\bigl\{ T_{t_{i+1}}f(x)-T_{t_{i}}f(x)\bigr\} _{\beta=\{ \epsilon_{i}\}\in\Theta}. \end{aligned}$$

Then

$$\begin{aligned} \mathcal{V}_{\rho}(T)f(x)= \bigl\Vert \mathcal{V}(T)f(x) \bigr\Vert _{F_{\rho}}. \end{aligned}$$

Next, let *B* be a Banach space and *φ* be a *B*-valued function, we define the sharp maximal operator as follows:

$$\begin{aligned} \varphi^{\sharp}(x)=\sup_{x\in I}\frac{1}{ \vert I \vert } \int_{I} \biggl\Vert \varphi (y)-\frac{1}{ \vert I \vert } \int_{I}\varphi(z)\,dz \biggr\Vert _{B}\,dy \thickapprox\sup_{ x\in I}\inf_{c} \frac{1}{ \vert I \vert } \int_{I} \bigl\Vert \varphi(y)-c \bigr\Vert _{B}\,dy. \end{aligned}$$

Then

$$\begin{aligned} M^{\sharp}\bigl(\mathcal{O}'(Tf)\bigr)\leq2\bigl( \mathcal{U}(T)f\bigr)^{\sharp}(x) \end{aligned}$$

and

$$\begin{aligned} M^{\sharp}\bigl(\mathcal{\mathcal{V}}_{\rho}(Tf)\bigr)\leq2\bigl( \mathcal {V}(T)f\bigr)^{\sharp}(x). \end{aligned}$$

Finally, let us recall some results about oscillation and variation operators.

#### Lemma 2.5

([5])

*Suppose that* $K(x,y)$ *satisfies* (1.1)-(1.3), $\rho >2$. *Let* $T=\{T_{\epsilon}\}_{\epsilon>0}$ *be given by* (1.4). *If* $O(T)$ *and* $V_{\rho}(T)$ *are bounded on* $L^{p_{0}}(R)$ *for some* $1< p_{0}<\infty$, *then*, *for any* $1< p<\infty$, $\omega\in A_{p}(\mathbb{R})$,

$$\begin{aligned} \bigl\Vert \mathcal{O}'(Tf) \bigr\Vert _{L^{p}(\omega)}\leq \bigl\Vert \mathcal{O}(Tf) \bigr\Vert _{L^{p}(\omega)}\lesssim \Vert f \Vert _{L^{p}(\omega)} \end{aligned}$$

*and*

$$\begin{aligned} \bigl\Vert \mathcal{V}_{\rho}(Tf) \bigr\Vert _{L^{p}(\omega)}\lesssim \Vert f \Vert _{L^{p}(\omega)}. \end{aligned}$$

## The proof of main results

Note that if $\omega\in A_{p,q}(\mathbb{R})$, then $\omega^{q}\in A_{\infty}(\mathbb{R})$. By Lemma 2.2 and Lemma 2.3, we only need to prove

3.1 $$\begin{aligned} M^{\sharp}\bigl(\mathcal{O}'\bigl(T^{b} \bigr)f\bigl)(x) \lesssim \bigl\Vert {b}^{(m)} \bigr\Vert _{\dot{\wedge }_{\beta}} \bigl( M_{\beta,r}(f) (x)+ M_{\beta}(f) (x) \bigr) \end{aligned}$$

and

3.2 $$\begin{aligned} M^{\sharp}\bigl(\mathcal{V}_{\rho}\bigl(T^{b} \bigr)f\bigl)(x) \lesssim \bigl\Vert {b}^{(m)} \bigr\Vert _{\dot {\wedge}_{\beta}} \bigl( M_{\beta,r}(f) (x)+ M_{\beta}(f) (x) \bigr) \end{aligned}$$

hold for any $1< r<\infty$.

We will prove only inequality (3.1), since (3.2) can be obtained by a similar argument. Fix *f* and $x_{0}$ with an interval $I=(x_{0}-l,x_{0}+l)$. Write $f=f_{1}+f_{2}=f\chi_{5I}+f\chi_{\mathbb{R}\setminus5I}$, and let

$$\begin{aligned} C_{I}= \biggl\{ \int_{\{t_{i+1}< \vert x_{0}-y \vert < s\}}\frac {R_{m+1}({b};x_{0},y)}{ \vert x_{0}-y \vert ^{m}}K(x_{0},y)f_{2}(y)\,dy \biggr\} _{s\in J_{i},i\in \mathbb{N}}=\mathcal{U}\bigl(T^{b}\bigr)f_{2}(x_{0}). \end{aligned}$$

Then

$$\begin{aligned} \mathcal{U}\bigl(T^{b}\bigr)f (x) ={}& \biggl\{ \int_{\{t_{i+1}< \vert x-y \vert < s\}}\frac {R_{m+1}({b};x,y)}{ \vert x-y \vert ^{m}}K(x,y)f(y)\,dy \biggr\} _{s\in J_{i},i\in\mathbb {N}} \\ ={}& \mathcal{U}(T) \biggl(\frac{R_{m+1}({b};x,\cdot)}{ \vert x-\cdot \vert ^{m}}f_{1} \biggr)+\mathcal{U} \bigl(T^{b}\bigr)f_{2}(x). \end{aligned}$$

Therefore

$$\begin{aligned} &\frac{1}{ \vert I \vert } \int_{I} \bigl\Vert \mathcal{U}\bigl(T^{b}\bigr)f (x)-C_{I} \bigr\Vert _{E}\,dx \\ &\quad \leq \frac{1}{ \vert I \vert } \int_{I} \biggl\Vert \mathcal{U}(T) \biggl( \frac {R_{m+1}({b};x,\cdot)}{ \vert x-\cdot \vert ^{m}}f_{1} \biggr) \biggr\Vert _{E}\,dx + \frac{1}{ \vert I \vert } \int_{I} \bigl\Vert \mathcal{U}\bigl(T^{b} \bigr)f_{2}(x)-\mathcal {U}\bigl(T^{b}\bigr)f_{2}(x_{0}) \bigr\Vert _{E}\,dx \\ &\quad = M_{1}+M_{2}. \end{aligned}$$

For $x\in I$, $k=0,-1,-2,\ldots$ , let $E_{k}=\{y:2^{k-1}\cdot6l\leq \vert y-x \vert <2^{k}\cdot6l\}$, let $I_{k}=\{y: \vert y-x \vert <2^{k}\cdot6l\}$, and let ${b}_{k}(z)=b(z)-\frac{1}{m!}(b^{(m)})_{I_{k}}z^{m}$. By [10] we have $R_{m+1}({b};x,y)=R_{m+1}(b_{k};x,y)$ for any $y\in E_{k}$.

By Lemma 2.5, we know $\mathcal{O}'(T)$ is bounded on $L^{u}(\mathbb{R})$ for $u>1$. Then, using Hölder’s inequality, we deduce

$$\begin{aligned} M_{1} \lesssim{}& \biggl(\frac{1}{ \vert I \vert } \int_{I} \biggl\Vert \mathcal{U}(T) \biggl( \frac {R_{m+1}({b};x,\cdot)}{ \vert x-\cdot \vert ^{m}}f_{1} \biggr) \biggr\Vert ^{u}_{E}\,dx \biggr)^{1/u} \\ \lesssim& \biggl(\frac{1}{ \vert I \vert } \int_{\{y: \vert y-x \vert < 6l\}} \biggl\vert \frac {R_{m+1}({b};\cdot,y)}{ \vert y-\cdot \vert ^{m}}f(y) \biggr\vert ^{u}\,dy \biggr)^{1/u} \\ =& \Biggl(\frac{1}{ \vert I \vert }\sum_{k=-\infty}^{0} \int_{E_{k}} \biggl\vert \biggl(\frac{R_{m+1}({b}_{k};\cdot,y)}{ \vert y-\cdot \vert ^{m}}f(y) \biggr) \biggr\vert ^{r}\,dy \Biggr)^{1/r} \\ \lesssim& \Biggl(\frac{1}{ \vert I \vert }\sum_{k=-\infty}^{0} \int_{E_{k}} \biggl\vert \biggl( \biggl(\frac{R_{m}({b_{k}};\cdot,y)}{ \vert y-\cdot \vert ^{m}}- \frac{1}{m!}\frac {(y-\cdot)^{m}{b}_{k}^{(m)}(y) }{ \vert y-\cdot \vert ^{m}} \biggr)f(y) \biggr) \biggr\vert ^{u}\,dy \Biggr)^{1/u} \\ \lesssim& \Biggl(\frac{1}{ \vert I \vert }\sum_{k=-\infty}^{0} \int_{E_{k}} \biggl\vert \frac{R_{m}({b_{k}};\cdot,y)}{ \vert y-\cdot \vert ^{m}}f(y) \biggr\vert ^{u}\,dy \Biggr)^{1/u} \\ &{} + \Biggl(\frac{1}{ \vert I \vert }\sum_{k=-\infty}^{0} \int_{E_{k}} \biggl\vert \frac {1}{m!} \frac{(y-\cdot)^{m}{b}_{k}^{(m)}(y) }{ \vert y-\cdot \vert ^{m}}f(y) \biggr\vert ^{u}\,dy \Biggr)^{1/u} \\ =& M_{11}+M_{12}. \end{aligned}$$

By Lemma 2.4 and Lemma 2.1,

$$\begin{aligned} \bigl\vert R_{m}({b}_{k};x,y) \bigr\vert \lesssim& \vert x-y \vert ^{m} \biggl(\frac{1}{ \vert I_{x}^{y} \vert } \int _{I_{x}^{y}} \bigl\vert {b}_{k}^{(m)}(z) \bigr\vert ^{s}\,dz \biggr)^{1/s} \\ \lesssim& \vert x-y \vert ^{m} \biggl(\frac{1}{2^{k}\cdot30l} \int _{ \vert y-x \vert < 2^{k}\cdot30l} \bigl\vert b^{(m)}(y)- \bigl(b^{(m)}\bigr)_{I_{k}} \bigr\vert ^{s}\,dz \biggr)^{1/s} \\ \lesssim& \vert x-y \vert ^{m}\bigl(2^{k}l \bigr)^{\beta}\bigl\Vert {b}^{(m)} \bigr\Vert _{\dot{\wedge}_{\beta}}. \end{aligned}$$

Then

$$\begin{aligned} M_{11} \lesssim& \bigl\Vert {b}^{(m)} \bigr\Vert _{\dot{\wedge}_{\beta}} l^{\beta}\Biggl(\frac{1}{ \vert I \vert }\sum _{k=-\infty}^{0}2^{k\beta u} \int _{E_{k}} \bigl\vert f(y) \bigr\vert ^{u}\,dy \Biggr)^{1/u} \\ \lesssim& \bigl\Vert {b}^{(m)} \bigr\Vert _{\dot{\wedge}_{\beta}} l^{\beta}\Biggl(\frac {1}{ \vert I \vert }\sum_{k=-\infty}^{0} \int_{E_{k}} \bigl\vert f(y) \bigr\vert ^{u}\,dy \Biggr)^{1/u} \\ \lesssim& \bigl\Vert {b}^{(m)} \bigr\Vert _{\dot{\wedge}_{\beta}} l^{\beta}\biggl(\frac {1}{ \vert I \vert } \int_{7I} \bigl\vert f(y) \bigr\vert ^{u}\,dy \biggr)^{1/u} \\ \lesssim& \bigl\Vert {b}^{(m)} \bigr\Vert _{\dot{\wedge}_{\beta}} l^{\beta}\biggl(\frac {1}{ \vert I \vert } \int_{7I} \bigl\vert f(y) \bigr\vert ^{r}\,dy \biggr)^{1/r} \\ \lesssim& \bigl\Vert {b}^{(m)} \bigr\Vert _{\dot{\wedge}_{\beta}} M_{\beta,r}(f) (x_{0}). \end{aligned}$$

Since ${b}_{k}^{(m)}(y)=b^{(m)}(y)-(b^{(m)})_{I_{k}}$, then, applying Hölder’s inequality and Lemma 2.1, we get

$$\begin{aligned} M_{12} \lesssim & \Biggl(\frac{1}{ \vert I \vert }\sum _{k=-\infty}^{0} \int _{E_{k}} \bigl\vert \bigl(b^{(m)}(y)- \bigl(b^{(m)}\bigr)_{I_{k}}\bigr)f(y) \bigr\vert ^{u}\,dy \Biggr)^{1/u} \\ \lesssim& \biggl(\frac{1}{ \vert I \vert }\sum_{k=-\infty}^{0} \biggl( \int _{I_{k}} \bigl\vert f(y) \bigr\vert ^{r}\,dy \biggr)^{u/r} \biggl( \int_{I_{k}} \bigl\vert b^{(m)}(y)- \bigl(b^{(m)}\bigr)_{I_{k}} \bigr\vert ^{\frac {ur}{r-u}} \biggr)^{1-u/r} \biggr)^{1/u} \\ \lesssim& \bigl\Vert {b}^{(m)} \bigr\Vert _{\dot{\wedge}_{\beta}} \Biggl( \frac {1}{ \vert I \vert }\sum_{k=-\infty}^{0} \biggl( \int_{I_{k}} \bigl\vert f(y) \bigr\vert ^{r}\,dy \biggr)^{u/r} \vert I_{k} \vert ^{\beta u+1-u/r} \Biggr)^{1/u} \\ \lesssim& \bigl\Vert {b}^{(m)} \bigr\Vert _{\dot{\wedge}_{\beta}} M_{\beta ,r}(f) (x_{0}) \Biggl(\frac{1}{ \vert I \vert }\sum _{k=-\infty}^{0} \vert I_{k} \vert \Biggr)^{1/u} \\ \lesssim& \bigl\Vert {b}^{(m)} \bigr\Vert _{\dot{\wedge}_{\beta}} M_{\beta,r}(f) (x_{0}). \end{aligned}$$

We now estimate $M_{2}$. For $x\in I$, we have

$$\begin{aligned} & \bigl\Vert \mathcal{U}\bigl(T^{b}\bigr)f_{2}(x)- \mathcal{U}\bigl(T^{b}\bigr)f_{2}(x_{0}) \bigr\Vert _{E} \\ &\quad = \biggl\Vert \biggl\{ \int_{\{t_{i+1}< \vert x-y \vert < s\}}\frac {R_{m+1}({b};x,y)}{ \vert x-y \vert ^{m}}K(x,y)f_{2}(y)\,dy \\ &\qquad{}- \int_{\{t_{i+1}< \vert x_{0}-y \vert < s\}}\frac {R_{m+1}({b};x_{0},y)}{ \vert x_{0}-y \vert ^{m}}K(x_{0},y)f_{2}(y)\,dy \biggr\} _{s\in J_{i}, i\in\mathbb{N}} \biggr\Vert _{E} \\ &\quad \leq \biggl\Vert \biggl\{ \int_{\{t_{i+1}< \vert x-y \vert < s\}} \biggl(\frac {R_{m+1}({b};x,y)}{ \vert x-y \vert ^{m}}K(x,y)- \frac{R_{m+1}({b};x_{0},y)}{ \vert x_{0}-y \vert ^{m}}K(x_{0},y) \biggr)f_{2}(y)\,dy\biggr\} _{s\in J_{i}, i\in\mathbb{N}} \biggr\Vert _{E} \\ &\qquad{} + \biggl\Vert \biggl\{ \int_{R} \bigl(\chi_{\{t_{i+1}< \vert x-y \vert < s\}}(y)-\chi _{\{t_{i+1}< \vert x_{0}-y \vert < s\}}(y) \bigr)\frac {R_{m+1}({b};x_{0},y)}{ \vert x_{0}-y \vert ^{m}}K(x_{0},y)f_{2}(y)\,dy \biggr\} _{s\in J_{i}, i\in \mathbb{N}} \biggr\Vert _{E} \\ &\quad = N_{1}+N_{2}. \end{aligned}$$

For $k=0,1,2,\ldots$ , let $F_{k}=\{y:2^{k}\cdot4l\leq \vert y-x_{0} \vert <2^{k+1}\cdot4l\}$, let $\widetilde{I}_{k}=\{y: \vert y-x_{0} \vert <2^{k}\cdot4l\}$, and let $\widetilde{b}_{k}(z)=b(z)-\frac {1}{m!}(b^{(m)})_{\widetilde{I}_{k}}z^{m}$. Note that

$$\begin{aligned} & \frac{R_{m+1}({b};x,y)}{ \vert x-y \vert ^{m}}K(x,y) -\frac{R_{m+1}({b};x_{0},y)}{ \vert x_{0}-y \vert ^{m}}K(x_{0},y) \\ &\quad =\frac{R_{m+1}(\widetilde{b}_{k};x,y)}{ \vert x-y \vert ^{m}}K(x,y) -\frac{R_{m+1}(\widetilde{b}_{k};x_{0},y)}{ \vert x_{0}-y \vert ^{m}}K(x_{0},y) \\ &\quad = \frac{1}{ \vert x-y \vert ^{m}} \bigl(R_{m}(\widetilde {b}_{k};x,y)-R_{m}( \widetilde{b}_{k};x_{0},y) \bigr)K(x,y) \\ &\qquad{} +R_{m}(\widetilde{b}_{k};x_{0},y) \biggl( \frac{1}{ \vert x-y \vert ^{m}}-\frac {1}{ \vert x_{0}-y \vert ^{m}} \biggr)K(x,y) \\ &\qquad{} - \frac{1}{m!}\widetilde{b}_{k}^{(m)}(y) \biggl( \frac {(x-y)^{m}}{ \vert x-y \vert ^{m}}-\frac{(x_{0}-y)^{m}}{ \vert x_{0}-y \vert ^{m}} \biggr)K(x,y) \\ &\qquad{} + \frac{R_{m+1}(\widetilde{b}_{k};x_{0},y)}{ \vert x_{0}-y \vert ^{m}} \bigl(K(x,y)-K(x_{0},y) \bigr). \end{aligned}$$

By Minkowski’s inequalities and $\Vert \{\chi_{\{ t_{i+1}< \vert x-y \vert <s\}}\}_{s\in J_{i}, i\in\mathbb{N}} \Vert _{E}\leq1$, we obtain

$$\begin{aligned} N_{1}\leq{}& \int_{\mathbb{R}} \bigl\Vert \{\chi_{\{t_{i+1}< \vert x-y \vert < s\} } \}_{s\in J_{i}, i\in\mathbb{N}} \bigr\Vert _{E} \\ &{}\times \biggl\vert \frac{R_{m+1}(\widetilde{b}_{k};x,y)}{ \vert x-y \vert ^{m}}K(x,y) -\frac{R_{m+1}(\widetilde{b}_{k};x_{0},y)}{ \vert x_{0}-y \vert ^{m}}K(x_{0},y) \biggr\vert \bigl\vert f_{2}(y) \bigr\vert \,dy \\ \leq{}& \sum _{k=0}^{\infty}\int_{F_{k}}\frac{1}{ \vert x-y \vert ^{m}} \bigl\vert R_{m}( \widetilde{b}_{k};x,y)-R_{m}(\widetilde{b}_{k};x_{0},y) \bigr\vert \bigl\vert K(x,y) \bigr\vert \bigl\vert f_{2}(y) \bigr\vert \,dy \\ &{}+ \sum_{k=0}^{\infty}\int_{F_{k}} \bigl\vert R_{m}(\widetilde{b}_{k};x_{0},y) \bigr\vert \biggl\vert \frac{1}{ \vert x-y \vert ^{m}}-\frac{1}{ \vert x_{0}-y \vert ^{m}} \biggr\vert \bigl\vert K(x,y) \bigr\vert \bigl\vert f_{2}(y) \bigr\vert \,dy \\ &{}+ \sum_{k=0}^{\infty}\int_{F_{k}}\frac{1}{m!} \bigl\vert \widetilde {b}_{k}^{(m)}(y) \bigr\vert \biggl\vert \frac{(x-y)^{m}}{ \vert x-y \vert ^{m}}-\frac {(x_{0}-y)^{m}}{ \vert x_{0}-y \vert ^{m}} \biggr\vert \bigl\vert K(x,y) \bigr\vert \bigl\vert f_{2}(y) \bigr\vert \,dy \\ &{}+ \sum_{k=0}^{\infty}\int_{F_{k}} \biggl\vert \frac{R_{m+1}(\widetilde {b}_{k};x_{0},y)}{ \vert x_{0}-y \vert ^{m}} \biggr\vert \bigl\vert K(x,y)-K(x_{0},y) \bigr\vert \bigl\vert f_{2}(y) \bigr\vert \,dy \\ ={}& N_{11}+N_{12}+N_{13}+N_{14}. \end{aligned}$$

From the mean value theorem, there exists $\eta\in I$ such that

$$\begin{aligned} R_{m}(\widetilde{b}_{k};x,y)-R_{m}(\widetilde {b}_{k};x_{0},y)=(x-x_{0})R_{m-1}\bigl( \widetilde{b}_{k}';\eta,y\bigr). \end{aligned}$$

For $\eta, x\in I$, $y\in F_{k}$, we have $\vert y-x_{0} \vert \thickapprox \vert y-x \vert \thickapprox \vert y-\eta \vert $ and $5 \vert y-\eta \vert \approx5 \vert y-x_{0} \vert \leq 2^{k+1}\cdot20l$. By Lemma 2.4 and Lemma 2.1 we get

$$\begin{aligned} \bigl\vert R_{m-1}\bigl(\widetilde{b}'_{k}; \eta,y\bigr) \bigr\vert \lesssim{}& \vert \eta-y \vert ^{m-1} \biggl( \frac{1}{ \vert I_{\eta}^{y} \vert } \int_{I_{\eta}^{y}} \bigl\vert \widetilde {b}_{k}^{(m)}(z) \bigr\vert ^{s}\,dz \biggr)^{1/s} \\ \lesssim{}& \vert x-y \vert ^{m-1} \biggl(\frac{1}{2^{k+1}\cdot20l} \int _{ \vert z-x_{0} \vert < 2^{k+1}\cdot20l} \bigl\vert {b}^{(m)}(z)- \bigl(b^{(m)}\bigr)_{\widetilde {I}_{k}} \bigr\vert ^{s}\,dz \biggr)^{1/s} \\ \lesssim{}& \bigl\Vert {b}^{(m)} \bigr\Vert _{\dot{\wedge}_{\beta}} \bigl(2^{k}l\bigr)^{\beta} \vert x-y \vert ^{m-1}. \end{aligned}$$

Then

$$\begin{aligned} \bigl\vert R_{m}(\widetilde{b}_{k};x,y)-R_{m}( \widetilde{b}_{k};x_{0},y) \bigr\vert \lesssim \bigl\Vert {b}^{(m)} \bigr\Vert _{\dot{\wedge}_{\beta}}\bigl(2^{k}l \bigr)^{\beta} \vert x-x_{0} \vert \vert x-y \vert ^{m-1}. \end{aligned}$$

Since $\vert K(x,y) \vert \leq C \vert x_{0}-y \vert ^{-1}$,

$$\begin{aligned} N_{11}\lesssim{}& \bigl\Vert {b}^{(m)} \bigr\Vert _{\dot{\wedge}_{\beta}}\sum_{k=0}^{\infty}\bigl(2^{k}l\bigr)^{\beta}\int_{2^{k}\cdot4l\leq \vert x_{0}-y \vert < 2^{k+1}\cdot4l}\frac{l}{(2^{k}\cdot4l)^{2}} \bigl\vert f(y) \bigr\vert \,dy \\ \lesssim{}& \bigl\Vert {b}^{(m)} \bigr\Vert _{\dot{\wedge}_{\beta}}\sum _{k=0}^{\infty}\frac{1}{2^{k}} \frac{(2^{k}l)^{\beta}}{2^{k}l} \int_{ \vert x_{0}-y \vert < 2^{k+1}\cdot4l} \bigl\vert f(y) \bigr\vert \,dy \\ \lesssim{}& \bigl\Vert {b}^{(m)} \bigr\Vert _{\dot{\wedge}_{\beta}}M_{\beta}(f) (x_{0}). \end{aligned}$$

For $N_{12}$, since $x\in I$, $y\in F_{k}$,

$$\begin{aligned} \bigl\vert R_{m}(\widetilde{b}_{k};x,y) \bigr\vert \lesssim \vert x-y \vert ^{m} \biggl(\frac {1}{ \vert I_{x}^{y} \vert } \int_{I_{x}^{y}} \bigl\vert \widetilde{b}_{k}^{(m)}(z) \bigr\vert ^{s}\,dz \biggr)^{1/s}\lesssim \bigl\Vert {b}^{(m)} \bigr\Vert _{\dot{\wedge}_{\beta}}\bigl(2^{k}l \bigr)^{\beta} \vert x-y \vert ^{m} \end{aligned}$$

and

$$\begin{aligned} \biggl\vert \frac{1}{ \vert x-y \vert ^{m}}-\frac{1}{ \vert x_{0}-y \vert ^{m}} \biggr\vert \lesssim \frac { \vert x-x_{0} \vert }{ \vert x-y \vert ^{m+1}}. \end{aligned}$$

Thus

$$\begin{aligned} N_{12}\lesssim \bigl\Vert {b}^{(m)} \bigr\Vert _{\dot{\wedge}_{\beta}}\sum_{k=0}^{\infty}\bigl(2^{k}l\bigr)^{\beta}\int_{2^{k}\cdot4l\leq \vert x_{0}-y \vert < 2^{k+1}\cdot4l}\frac {l}{(2^{k}\cdot4l)^{2}} \bigl\vert f(y) \bigr\vert \,dy \lesssim \bigl\Vert {b}^{(m)} \bigr\Vert _{\dot{\wedge }_{\beta}}M_{\beta}(f) (x_{0}). \end{aligned}$$

As for $N_{13}$, due to

$$\begin{aligned} \biggl\vert \frac{(x-y)^{m}}{ \vert x-y \vert ^{m}}-\frac{(x_{0}-y)^{m}}{ \vert x_{0}-y \vert ^{m}} \biggr\vert \lesssim \frac{ \vert x-x_{0} \vert }{ \vert x-y \vert }, \end{aligned}$$

and noting $\widetilde{b}_{k}^{(m)}(y)=b^{(m)}(y)-(b^{(m)})_{\widetilde {I}_{k}}$, we have

$$\begin{aligned} N_{13}\lesssim{}& \sum_{k=0}^{\infty}\int_{F_{k}} \bigl\vert b^{(m)}(y)- \bigl(b^{(m)}\bigr)_{\widetilde{I}_{k}} \bigr\vert \frac { \vert x-x_{0} \vert }{ \vert x_{0}-y \vert ^{2}} \bigl\vert f(y) \bigr\vert \,dy \\ \lesssim{}& \sum_{k=0}^{\infty}\frac{1}{2^{k}}\frac{1}{2^{k}\cdot 4l} \int_{ \vert x_{0}-y \vert < 2^{k}\cdot4l} \bigl\vert b^{(m)}(y)- \bigl(b^{(m)}\bigr)_{\widetilde {I}_{k}} \bigr\vert \bigl\vert f(y) \bigr\vert \,dy \\ \lesssim{}& \sum_{k=0}^{\infty}\frac{1}{2^{k}} \biggl(\frac {1}{2^{k}\cdot4l} \int_{ \vert x_{0}-y \vert < 2^{k}\cdot4l} \bigl\vert f(y) \bigr\vert ^{r}\,dy \biggr)^{1/r} \\ &{} \times \biggl(\frac{1}{2^{k}\cdot4l} \int_{ \vert x_{0}-y \vert < 2^{k}\cdot 4l} \bigl\vert b^{(m)}(y)- \bigl(b^{(m)}\bigr)_{\widetilde{I}_{k}} \bigr\vert ^{r'}\,dy \biggr)^{1/r'} \\ \lesssim{}& \bigl\Vert {b}^{(m)} \bigr\Vert _{\dot{\wedge}_{\beta}}M_{r,\beta }(f) (x_{0})\sum_{k=0}^{\infty}\frac{1}{2^{k}}\lesssim \bigl\Vert {b}^{(m)} \bigr\Vert _{\dot {\wedge}_{\beta}}M_{\beta,r}(f) (x_{0}). \end{aligned}$$

Notice

$$\begin{aligned} \bigl\vert R_{m+1}(\widetilde{b}_{k}; x_{0},y) \bigr\vert &\leq \bigl\vert R_{m}(\widetilde {b}_{k};x_{0},y) \bigr\vert +\frac{1}{m!} \bigl\vert \widetilde{b}_{k}^{(m)}(y) (x_{0}-y)^{m} \bigr\vert \\ & \lesssim \bigl\Vert b^{(m)} \bigr\Vert _{\dot{\wedge}_{\beta}} \bigl(2^{k}l\bigr)^{\beta} \vert x_{0}-y \vert ^{m}+ \bigl\vert b^{(m)}(y)-\bigl(b^{(m)} \bigr)_{\widetilde{I}_{k}} \bigr\vert \vert x_{0}-y \vert ^{m} \end{aligned}$$

and by (1.2),

$$\begin{aligned} \bigl\vert K(x,y)-K(x_{0},y) \bigr\vert \lesssim \frac{ \vert x-x_{0} \vert ^{\delta}}{ \vert x_{0}-y \vert ^{1+\delta}}. \end{aligned}$$

Similar to the estimates for $N_{11}$, we have

$$\begin{aligned} \sum_{k=0}^{\infty}\int_{F_{k}}\frac{ \vert R_{m}(\widetilde {b}_{k};x_{0},y) \vert }{ \vert x-y \vert ^{m}}\frac{ \vert x-x_{0} \vert ^{\delta}}{ \vert x_{0}-y \vert ^{1+\delta }} \bigl\vert f(y) \bigr\vert \,dy\lesssim \bigl\Vert {b}^{(m)} \bigr\Vert _{\dot{\wedge}_{\beta}}M_{\beta}(f) (x_{0}). \end{aligned}$$

Similar to the estimates for $N_{13}$, we have

$$\begin{aligned} \sum_{k=0}^{\infty}\int_{F_{k}}\frac{ \vert \widetilde {b}_{k}^{(m)}(y)(x_{0}-y)^{m} \vert }{ \vert x-y \vert ^{m}}\frac{ \vert x-x_{0} \vert ^{\delta}}{ \vert x_{0}-y \vert ^{1+\delta}} \bigl\vert f(y) \bigr\vert \,dy\lesssim \bigl\Vert {b}^{(m)} \bigr\Vert _{\dot{\wedge }_{\beta}}M_{\beta,r}(f) (x_{0}). \end{aligned}$$

Then

$$\begin{aligned} N_{14}\lesssim \bigl\Vert {b}^{(m)} \bigr\Vert _{\dot{\wedge}_{\beta}} \bigl(M_{\beta}(f) (x_{0})+M_{\beta,r}(f) (x_{0}) \bigr). \end{aligned}$$

Finally, let us estimate $N_{2}$. Notice that the integral

$$\begin{aligned} \int_{R} \bigl(\chi_{\{t_{i+1}< \vert x-y \vert < s\}}(y)-\chi_{\{ t_{i+1}< \vert x_{0}-y \vert < s\}}(y) \bigr) \frac{R_{m+1}({b};x_{0},y)}{ \vert x_{0}-y \vert ^{m}}K(x_{0},y)f_{2}(y)\,dy \end{aligned}$$

will be non-zero in the following cases:

(i) $t_{i+1}< \vert x-y \vert <s$ and $\vert x_{0}-y \vert \leq t_{i+1}$;
(ii) $t_{i+1}< \vert x-y \vert <s$ and $\vert x_{0}-y \vert \geq s$;
(iii) $t_{i+1}< \vert x_{0}-y \vert <s$ and $\vert x-y \vert \leq t_{i+1}$;
(iv) $t_{i+1}< \vert x_{0}-y \vert <s$ and $\vert x-y \vert \geq s$.

In case (i) we have $t_{i+1}< \vert x-y \vert \leq \vert x_{0}-x \vert + \vert x_{0}-y \vert <l+t_{i+1}$ as $\vert x-x_{0} \vert < l$. Similarly, in case (iii) we have $t_{i+1}< \vert x_{0}-y \vert <l+t_{i+1}$ as $\vert x-x_{0} \vert < l$. In case (ii) we have $s< \vert x_{0}-y \vert <l+s$ and in case (iv) we have $s< \vert x-y \vert <l+s$. By (1.1) and taking $1< t< r$, we have

$$\begin{aligned} &\int_{\mathbb{R}} \bigl(\chi_{\{t_{i+1}< \vert x-y \vert < s\}}(y)-\chi_{\{ t_{i+1}< \vert x_{0}-y \vert < s\}}(y) \bigr) \frac{R_{m+1}({b};x_{0},y)}{ \vert x_{0}-y \vert ^{m}}K(x_{0},y)f_{2}(y)\,dy \\ &\quad \lesssim \int_{\mathbb{R}}\chi_{\{t_{i+1}< \vert x-y \vert < s\}}(y)\chi_{\{ t_{i+1}< \vert x-y \vert < l+t_{i+1}\}}(y) \biggl\vert \frac{R_{m+1}({b};x_{0},y)}{ \vert x_{0}-y \vert ^{m}} \biggr\vert \frac { \vert f_{2}(y) \vert }{ \vert x_{0}-y \vert }\,dy \\ &\qquad{} + \int_{\mathbb{R}}\chi_{\{t_{i+1}< \vert x-y \vert < s\}}(y)\chi_{\{ s< \vert x_{0}-y \vert < l+s\}}(y) \biggl\vert \frac{R_{m+1}({b};x_{0},y)}{ \vert x_{0}-y \vert ^{m}} \biggr\vert \frac { \vert f_{2}(y) \vert }{ \vert x_{0}-y \vert }\,dy \\ &\qquad{} + \int_{\mathbb{R}}\chi_{\{t_{i+1}< \vert x_{0}-y \vert < s\}}(y)\chi_{\{ t_{i+1}< \vert x_{0}-y \vert < l+t_{i+1}\}}(y) \biggl\vert \frac{R_{m+1}({b};x_{0},y)}{ \vert x_{0}-y \vert ^{m}} \biggr\vert \frac { \vert f_{2}(y) \vert }{ \vert x_{0}-y \vert }\,dy \\ &\qquad{} + \int_{\mathbb{R}}\chi_{\{t_{i+1}< \vert x_{0}-y \vert < s\}}(y)\chi_{\{ s< \vert x-y \vert < l+s\}}(y) \biggl\vert \frac{R_{m+1}({b};x_{0},y)}{ \vert x_{0}-y \vert ^{m}} \biggr\vert \frac { \vert f_{2}(y) \vert }{ \vert x_{0}-y \vert }\,dy \\ &\quad \lesssim l^{1/t'} \biggl( \int_{\mathbb{R}}\chi_{\{ t_{i+1}< \vert x-y \vert < s\}}(y) \biggl\vert \frac{R_{m+1}({b};x_{0},y)}{ \vert x_{0}-y \vert ^{m}} \biggr\vert ^{t} \frac{ \vert f_{2}(y) \vert ^{t}}{ \vert x_{0}-y \vert ^{t}}\,dy \biggr)^{1/t} \\ &\qquad{} + l^{1/t'} \biggl( \int_{\mathbb{R}}\chi_{\{t_{i+1}< \vert x_{0}-y \vert < s\} }(y) \biggl\vert \frac{R_{m+1}({b};x_{0},y)}{ \vert x_{0}-y \vert ^{m}} \biggr\vert ^{t} \frac{ \vert f_{2}(y) \vert ^{t}}{ \vert x_{0}-y \vert ^{t}}\,dy \biggr)^{1/t}. \end{aligned}$$

Then

$$\begin{aligned} N_{2}\lesssim{}& l^{1/t'} \biggl\Vert \biggl\{ \biggl( \int_{\mathbb{R}}\chi _{\{t_{i+1}< \vert x-y \vert < s\}}(y) \biggl\vert \frac {R_{m+1}({b};x_{0},y)}{ \vert x_{0}-y \vert ^{m}} \biggr\vert ^{t} \frac{ \vert f_{2}(y) \vert ^{t}}{ \vert x_{0}-y \vert ^{t}}\,dy \biggr)^{1/t}\biggr\} _{s\in J_{i}, i\in \mathbb{N}} \biggr\Vert _{E} \\ &{} +l^{1/t'} \biggl\Vert \biggl\{ \biggl( \int_{\mathbb{R}}\chi_{\{ t_{i+1}< \vert x_{0}-y \vert < s\}}(y) \biggl\vert \frac {R_{m+1}({b};x_{0},y)}{ \vert x_{0}-y \vert ^{m}} \biggr\vert ^{t} \frac{ \vert f_{2}(y) \vert ^{t}}{ \vert x_{0}-y \vert ^{t}}\,dy \biggr)^{1/t}\biggr\} _{s\in J_{i}, i\in \mathbb{N}} \biggr\Vert _{E} \\ ={}& N_{21}+N_{22}. \end{aligned}$$

Notice

$$\begin{aligned} \bigl\vert R_{m+1}(\widetilde{b}_{k};x_{0},y) \bigr\vert \lesssim \bigl\Vert b^{(m)} \bigr\Vert _{\dot{\wedge}_{\beta}} \bigl(2^{k}l\bigr)^{\beta} \vert x_{0}-y \vert ^{m}+ \bigl\vert b^{(m)}(y)-\bigl(b^{(m)} \bigr)_{\widetilde{I}_{k}} \bigr\vert \vert x_{0}-y \vert ^{m}. \end{aligned}$$

Choosing $1< r< p$ with $t=\sqrt{r}$, we have

$$\begin{aligned} N_{21}\lesssim{}& l^{1/t'}\biggl\{ \sum _{i\in\mathbb{N}}\sup_{s\in J_{i}} \biggl( \int_{\mathbb{R}}\chi_{\{t_{i+1}< \vert x-y \vert < s\}}(y) \biggl\vert \frac{R_{m+1}({b};x_{0},y)}{ \vert x_{0}-y \vert ^{m}} \biggr\vert ^{t} \frac{ \vert f_{2}(y) \vert ^{t}}{ \vert x_{0}-y \vert ^{t}}\,dy \biggr)^{2/t}\biggr\} ^{1/2} \\ \lesssim{}& l^{1/t'}\biggl\{ \sum_{i\in\mathbb{N}} \int_{\mathbb {R}}\chi_{\{t_{i+1}< \vert x-y \vert < t_{i}\}}(y) \biggl\vert \frac {R_{m+1}({b};x_{0},y)}{ \vert x_{0}-y \vert ^{m}} \biggr\vert ^{t} \frac{ \vert f_{2}(y) \vert ^{t}}{ \vert x_{0}-y \vert ^{t}}\,dy\biggr\} ^{1/t} \\ \lesssim{}& l^{1/t'}\biggl\{ \int_{\mathbb{R}} \biggl\vert \frac {R_{m+1}({b};x_{0},y)}{ \vert x_{0}-y \vert ^{m}} \biggr\vert ^{t} \frac{ \vert f_{2}(y) \vert ^{t}}{ \vert x_{0}-y \vert ^{t}}\,dy\biggr\} ^{1/t} \\ \lesssim{}& l^{1/t'}\Biggl\{ \sum_{k=0}^{\infty}\int_{F_{k}} \biggl\vert \frac {R_{m+1}(\widetilde{b}_{k};x_{0},y)}{ \vert x_{0}-y \vert ^{m}} \biggr\vert ^{t} \frac{ \vert f_{2}(y) \vert ^{t}}{ \vert x_{0}-y \vert ^{t}}\,dy\Biggr\} ^{1/t} \\ \lesssim{}& \bigl\Vert {b}^{(m)} \bigr\Vert _{\dot{\wedge}_{\beta}}l^{1/t'} \Biggl\{ \sum_{k=0}^{\infty}\bigl(2^{k}l \bigr)^{\beta t} \int_{F_{k}}\frac { \vert f(y) \vert ^{t}}{ \vert x_{0}-y \vert ^{t}}\,dy\Biggr\} ^{1/t} \\ &{} +l^{1/t'}\Biggl\{ \sum_{k=0}^{\infty}\int_{F_{k}} \bigl( \bigl\vert b^{(m)}(y)- \bigl(b^{(m)}\bigr)_{\widetilde{I}_{k}} \bigr\vert \bigr)^{t} \frac{ \vert f(y) \vert ^{t}}{ \vert x_{0}-y \vert ^{t}}\,dy\Biggr\} ^{1/t}. \end{aligned}$$

But

$$\begin{aligned} & l^{1/t'}\Biggl\{ \sum_{k=0}^{\infty}\bigl(2^{k}l\bigr)^{\beta t} \int_{F_{k}}\frac { \vert f(y) \vert ^{t}}{ \vert x_{0}-y \vert ^{t}}\,dy\Biggr\} ^{1/t} \\ &\quad \lesssim l^{1/t'} \Biggl(\sum_{k=1}^{\infty}\frac{(2^{k}l)^{\beta t}}{(2^{k}\cdot4l)^{t}} \int_{ \vert x_{0}-y \vert < 2^{k+1}\cdot4l} \bigl\vert f(y) \bigr\vert ^{t}\,dy \Biggr)^{1/t} \\ &\quad \lesssim \Biggl(\sum_{k=1}^{\infty}\frac{1}{2^{k(t-1)}}\frac {(2^{k}l)^{\beta t}}{2^{k}\cdot5l} \int_{ \vert x_{0}-y \vert < 2^{k}\cdot 5l} \bigl\vert f(y) \bigr\vert ^{t}\,dy \Biggr)^{1/t} \\ &\quad \lesssim \Biggl(\sum_{k=1}^{\infty}\frac{1}{2^{k(t-1)}} \biggl(\frac {(2^{k}l)^{\beta t^{2}}}{2^{k}\cdot5l} \int_{ \vert x_{0}-y \vert < 2^{k}\cdot 5l} \bigl\vert f(y) \bigr\vert ^{t^{2}}\,dy \biggr)^{1/t} \Biggr)^{1/t} \\ &\quad \lesssim \Biggl(\sum_{k=1}^{\infty}\frac{1}{2^{k(t-1)}} \Biggr)^{1/t}M_{\beta,r}(f) (x_{0}) \lesssim M_{\beta,r}(f) (x_{0}) \end{aligned}$$

and

$$\begin{aligned} & l^{1/t'}\Biggl\{ \sum_{k=0}^{\infty}\int_{F_{k}} \bigl( \bigl\vert b^{(m)}(y)- \bigl(b^{(m)}\bigr)_{\widetilde{I}_{k}} \bigr\vert \bigr)^{t} \frac{ \vert f(y) \vert ^{t}}{ \vert x_{0}-y \vert ^{t}}\,dy\Biggr\} ^{1/t} \\ &\quad \lesssim \Biggl(\sum_{k=0}^{\infty}\frac{1}{2^{k(t-1)}}\frac {1}{2^{k}\cdot4l} \int_{ \vert x_{0}-y \vert < 2^{k}\cdot 4l} \bigl\vert b^{(m)}(y)- \bigl(b^{(m)}\bigr)_{\widetilde{I}_{k}} \bigr\vert ^{t} \bigl\vert f(y) \bigr\vert ^{t}\,dy \Biggr)^{1/t} \\ &\quad \lesssim \Biggl(\sum_{k=0}^{\infty}\frac{1}{2^{k(t-1)}} \biggl(\frac {1}{2^{k}\cdot4l} \int_{ \vert x_{0}-y \vert < 2^{k}\cdot4l} \bigl\vert f(y) \bigr\vert ^{t^{2}}\,dy \biggr)^{1/t} \\ &\qquad{}\times \biggl(\frac{1}{2^{k}\cdot4l} \int_{ \vert x_{0}-y \vert < 2^{k}\cdot 4l} \bigl\vert b^{(m)}(y)- \bigl(b^{(m)}\bigr)_{\widetilde{I}_{k}} \bigr\vert ^{tt'} \biggr)^{1/t'} \Biggr)^{1/t} \\ &\quad \lesssim \bigl\Vert b^{(m)} \bigr\Vert _{\dot{\wedge}_{\beta}} \Biggl(\sum _{k=0}^{\infty}\frac{1}{2^{k(t-1)}} \biggl( \frac{(2^{k}\cdot4l)^{r\beta }}{2^{k}\cdot4l} \int_{ \vert x_{0}-y \vert < 2^{k}\cdot4l} \bigl\vert f(y) \bigr\vert ^{t^{2}}\,dy \biggr)^{1/t} \Biggr)^{1/t} \\ &\quad \lesssim \bigl\Vert b^{(m)} \bigr\Vert _{\dot{\wedge}_{\beta}} M_{\beta ,r}(f) (x_{0}) \Biggl(\sum_{k=0}^{\infty}\frac{1}{2^{k(t-1)}} \Biggr)^{1/t} \\ &\quad \lesssim \bigl\Vert b^{(m)} \bigr\Vert _{\dot{\wedge}_{\beta}} M_{\beta,r}(f) (x_{0}). \end{aligned}$$

Therefore

$$\begin{aligned} N_{21}\lesssim \bigl\Vert b^{(m)} \bigr\Vert _{\dot{\wedge}_{\beta}} M_{\beta,r}(f) (x_{0}). \end{aligned}$$

Similarly,

$$\begin{aligned} N_{22}\lesssim \bigl\Vert b^{(m)} \bigr\Vert _{\dot{\wedge}_{\beta}} M_{\beta,r}(f) (x_{0}). \end{aligned}$$

This completes the proof of (3.1). Hence, Theorem 1.1 is proved.
